# Recurrent Spasmodic Croup‐Like Episodes With Cough‐Predominant Symptoms in an Atopic School‐Aged Child: The Importance of Integrated Upper‐ and Lower‐Airway Management

**DOI:** 10.1002/rcr2.70686

**Published:** 2026-08-25

**Authors:** Rina Sakaguchi, Shizuka Mitsubuchi, Yusuke Miyashita, Toshihiko Nonaka, Kimitoshi Nakamura

**Affiliations:** ^1^ Department of Pediatrics Amakusa Medical Center Amakusa Japan; ^2^ Department of Pediatrics, Graduate School of Medical Sciences Kumamoto University Kumamoto Japan

**Keywords:** adherence, allergic rhinitis, asthma, recurrent spasmodic croup, united airway disease

## Abstract

Recurrent spasmodic croup is uncommon in school‐aged children and may reflect an atopy‐associated upper‐airway dysfunction rather than a viral illness. We reported a 14‐year‐old boy with perennial allergic rhinitis and cough‐predominant asthma who experienced 10 early‐morning episodes of barking cough and inspiratory stridor between 2020 and 2024. Despite the long‐term prescription of inhaled corticosteroid therapy, seasonal discontinuation and inconsistent adherence were evident, and recurrent episodes persisted. After referral, integrated management including stepwise allergic‐rhinitis treatment and re‐education promoting year‐round adherence was implemented. Although the direct impact of these interventions on episode recurrence could not be determined, cough symptoms improved and asthma control stabilized despite persistently elevated fractional nitric oxide (~60 ppb). Integrated upper‐ and lower‐airway management and adherence optimization may help stabilize cough‐predominant symptoms in atopic children.

## Graphical abstract

Clinical course from 2019 to 2026. Stars indicate croup episodes, the black polygons indicate cough burden, * indicates a period of increased cough burden. No recurrence of croup episodes was observed during follow‐up after February 2024. ICS, inhaled corticosteroid; INCS, intranasal corticosteroid; LABA, long‐acting β2‐agonist; LTRA, leukotriene receptor antagonist.

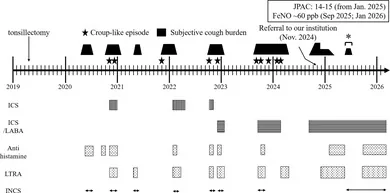

## 1 Introduction

Spasmodic croup is a non‐infectious form of croup characterized by the sudden onset of barking cough and inspiratory stridor, typically occurring at night or in the early morning, in the absence of fever [1]. Unlike viral croup, spasmodic croup is often recurrent and has been linked to atopic predisposition, cold air exposure and upper airway hyperreactivity [2]. Spasmodic croup typically decreases with age, but recurrent episodes over multiple seasons can pose diagnostic challenges, particularly in school aged children. Proposed risk factors for recurrence include asthma, allergic rhinitis and atopic dermatitis [3].

Children with recurrent spasmodic croup frequently present with overlapping respiratory symptoms, including chronic cough, exercise‐induced cough or nocturnal symptoms, leading to diagnostic overlap with asthma. As a result, management may focus on asthma‐directed therapy, whereas upper airway contributors such as allergic rhinitis and postnasal drip may remain underrecognized. We report the case of a child with recurrent spasmodic croup‐like episodes and prominent cough‐dominant symptoms. Enhanced management of allergic rhinitis together with more consistent year‐round airway control was associated with sustained stabilization of cough‐predominant symptoms and no recurrence of croup‐like episodes during follow‐up.

## 2 Case Report

**FIGURE 1:**
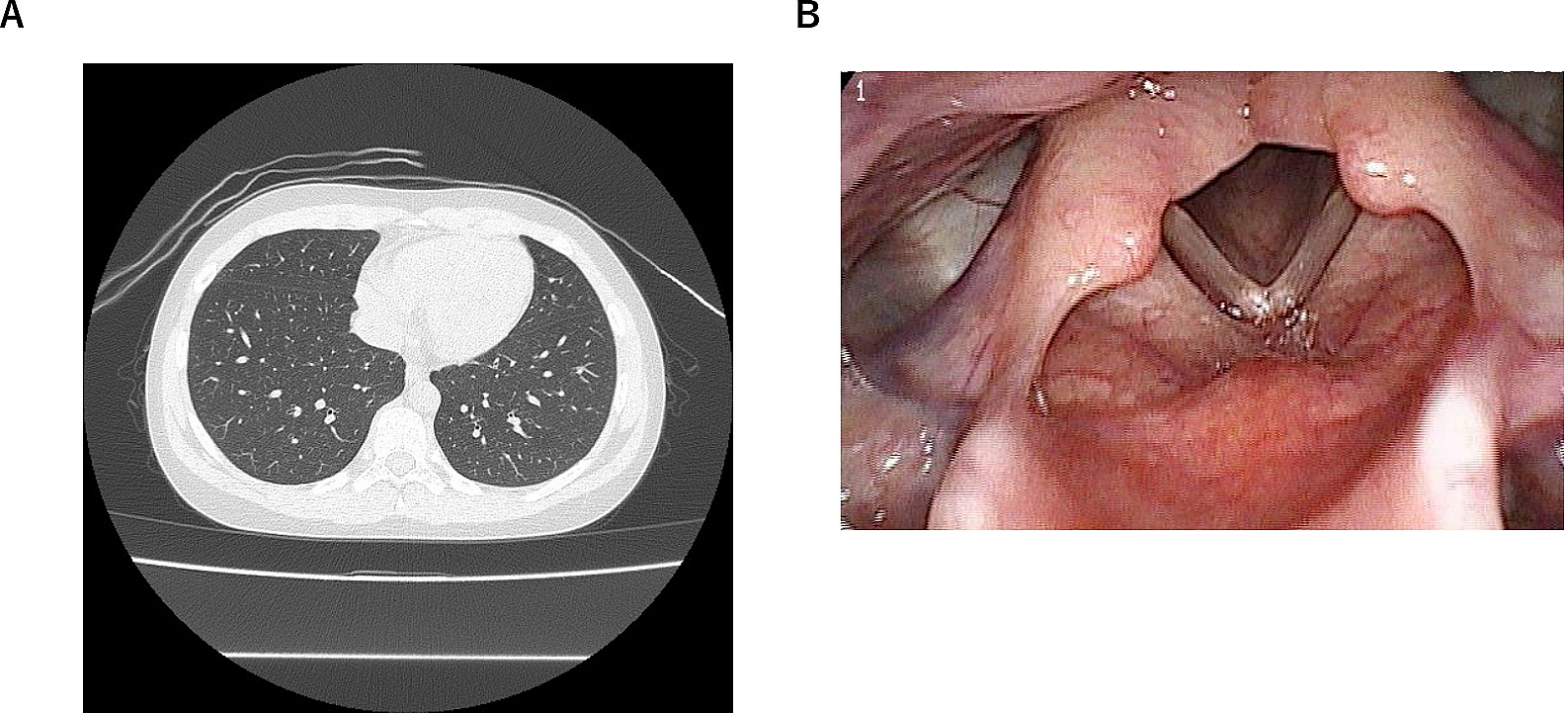
Chest computed tomography (A, March 2024) and Laryngoscopy (B, October 2023). Laryngoscopy was performed during asymptomatic and demonstrated no obstructive lesion in the upper airway.

The patient was a 14‐year‐old boy with perennial allergic rhinitis and cough predominant asthma. The patient had undergone tonsillectomy without any complications in 2019. Detailed records regarding the indication for surgery were unavailable. Beginning in the winter of 2020, he developed recurrent early morning episodes of sudden barking cough and inspiratory stridor, without fever or viral prodrome. According to caregiver reports, the episodes were accompanied by suprasternal retractions, and home pulse oximetry occasionally showed oxygen desaturation to approximately 85% during severe attacks. No hoarseness was reported. Each episode was treated with inhaled procaterol and inhaled budesonide as treatment for concomitant asthma symptoms, together with oral dexamethasone, which may have contributed to improvement of croup‐like symptoms. Overall symptoms improved substantially within 30–60 min. No hospitalizations or emergency department visits occurred. Ten episodes occurred between November 2020 and February 2024, showing clear autumn‐winter clustering. During this period, inhaled corticosteroid therapy was prescribed. However, the patient routinely discontinued controller medications from April to August, and adherence during winter was inconsistent. The highest seasonal clustering was observed between September 2023 and February 2024. During this interval, a multi month supply of inhalers reduced clinic visits, limiting opportunities to adjust concomitant allergic rhinitis therapy. This may have contributed to insufficient control of upper‐airway inflammation during the same interval. Chest computed tomography and laryngoscopy were performed, but no structural abnormalities explaining the cough or recurrent croup‐like episodes were identified (Figure 1).

He was referred to our institution in November 2024 for persistent cough and suboptimal asthma control. At presentation, he reported frequent early morning and exertional cough requiring repeated short acting β_2_ agonist use. Physical examination results were unremarkable. Allergen‐specific IgE testing using the fluorescence enzyme immunoassay (FEIA) method revealed strong sensitization to house dust mites (83.7 UA/mL) and cedar pollen (51.3 UA/mL), with elevated eosinophils (9.4%; 702/μL) and total IgE (699 IU/mL). He reported perennial nasal obstruction and rhinorrhea, worse in winter to spring, consistent with uncontrolled allergic rhinitis.

Stepwise allergic rhinitis management was initiated, including administration of a leukotriene receptor antagonist (November 2024), second‐generation antihistamines (January 2025), and continuous intranasal corticosteroids (from May 2025), followed by combination therapy from September 2025. Structured re‐education for patients and caregivers emphasized year‐round adherence to controller therapy. Periods of temporary discontinuation of oral anti‐allergic therapy were associated with subjective worsening of cough.

No further croup‐like episodes occurred after February 2024 despite the subsequent winter seasons. At referral in November 2024, asthma symptom control was retrospectively estimated to correspond to a JPAC (Japanese Pediatric Asthma Control) score of approximately 11 based on caregiver‐reported symptoms, whereas scores improved to 14–15 from January 2025 onward. Pulmonary function demonstrated no evidence of restrictive or obstructive ventilatory impairment (%VC: 87.3%, FEV1/FVC: 82.9%). Bronchodilator reversibility testing and bronchial provocation testing were not performed. However, cough symptoms showed favourable response to short‐acting β2‐agonists therapy. Fractional exhaled nitric oxide (FeNO) remained elevated at approximately 60 ppb (September 2025 and January 2026), suggesting persistent type 2 airway inflammation despite improved symptom control.

## 3 Discussion

This case illustrates recurrent croup‐like episodes may occur in the setting of overlapping upper‐ and lower‐ airway allergic diseases, including inadequately controlled allergic rhinitis and cough‐predominant asthma. Although recurrent spasmodic croup was recognized early, management focused on acute treatment and asthma directed therapy, whereas upper airway inflammation was not systematically addressed. This case also emphasizes the importance of reassessing adherence and seasonal discontinuation of controller therapy, as optimization through re‐education coincided with a substantial improvement in symptom control, consistent with guideline‐based asthma management [4, 5].

A key feature of this case was that recurrent seasonally clustered croup‐like episodes persisted despite repeated prescription of asthma controller therapy. However, interpretation of treatment efficacy was complicated by seasonal discontinuation and inconsistent adherence. Therefore, it remains difficult to determine whether persistence of symptoms reflected insufficient efficacy of asthma‐directed therapy itself or inadequate continuity of treatment. These findings emphasize the importance of adherence assessment together with comprehensive upper‐ and lower‐airway management in children with overlapping allergic airway diseases.

In this patient, FeNO remained elevated at approximately 60 ppb on two separate occasions (September 2025 and January 2026), despite marked improvement in symptom control [6]. This discordance suggests that type 2 airway inflammation may have persisted while the symptom burden was successfully stabilized. Several factors may have contributed to the persistently elevated FeNO levels, including ongoing allergic rhinitis, residual eosinophilic airway inflammation, and imperfect long‐term adherence despite repeated educational interventions. Peripheral blood eosinophilia also persisted during follow‐up, supporting the possibility of ongoing type 2 inflammation. These findings highlight that FeNO levels alone may not fully reflect the symptom burden in patients with coexisting allergic rhinitis and cough‐predominant respiratory symptoms.

Several limitations should be acknowledged. Objective assessment during acute episodes was unavailable because all evaluations, including laryngoscopy, were performed during asymptomatic periods. Therefore, inducible laryngeal obstruction or episodic vocal cord dysfunction could not be completely excluded. The diagnosis of recurrent spasmodic croup‐like episodes was based primarily on the characteristic clinical presentation based on caregiver reports.

This case is notable because recurrent spasmodic croup‐like episodes persisted into adolescence and occurred in the setting of seasonal discontinuation and inconsistent adherence to controller therapy, and coexisted with inadequately controlled allergic rhinitis. Furthermore, this case highlights the educational importance of integrated assessment and management of upper‐ and lower‐airway allergic diseases, particularly when cough‐predominant symptoms may lead clinicians to focus primarily on asthma control. Optimization of allergic rhinitis management together with reinforcement of consistent asthma controller use was associated with stabilization of cough symptoms. Although causality cannot be confirmed, the sustained improvement in cough‐predominant symptoms supports the importance of comprehensive airway assessment and long‐term management in children with overlapping upper‐ and lower‐airway disease. These features distinguish the present case from previously reported associations between recurrent croup and atopic disease.

## Author Contributions

Rina Sakaguchi, Shizuka Mitsubuchi, Toshihiko Nonaka and Yusuke Miyashita managed the patients. Rina Sakaguchi and Yusuke Miyashita contributed to writing the manuscript, and Kimitoshi Nakamura contributed to editing the manuscript. All the authors have read and approved the final version of the manuscript.

## Consent

The authors declare that written informed consent was obtained for the publication of this manuscript and accompanying images and attest that the form used to obtain consent from the patients complies with the Journal requirements as outlined in the author guidelines.

## Conflicts of Interest

The authors declare no conflicts of interest.

## Data Availability

Data sharing not applicable to this article as no datasets were generated or analyzed during the current study.
